# Discriminating between Different Heavy Metal Ions with Fullerene-Derived Nanoparticles

**DOI:** 10.3390/s18051496

**Published:** 2018-05-10

**Authors:** Erica Ciotta, Paolo Prosposito, Pietro Tagliatesta, Chiara Lorecchio, Lorenzo Stella, Saulius Kaciulis, Peiman Soltani, Ernesto Placidi, Roberto Pizzoferrato

**Affiliations:** 1 Department of Industrial Engineering, University of Rome Tor Vergata, 00133 Rome, Italy; Erica.Ciotta@uniroma2.it (E.C.); paolo.prosposito@uniroma2.it (P.P.); 2 INSTM and CiMER, University of Rome Tor Vergata, 00133 Rome, Italy; 3 Department of Chemical Sciences and Technology, University of Rome Tor Vergata, 00133 Rome, Italy; pietro.tagliatesta@uniroma2.it (P.T.); chiaralorec@hotmail.it (C.L.); stella@stc.uniroma2.it (L.S.); 4 Institute for the Study of Nanostructured Materials, CNR of Italy, Monterotondo Stazione, 00015 Rome, Italy; saulius.kaciulis@ismn.cnr.it (S.K.); peymansoltani14@gmail.com (P.S.); 5 CNR—Istituto di Struttura della Materia, Via Fosso del Cavaliere 100, 00133 Roma, Italy; ernesto.placidi@roma2.infn.it

**Keywords:** optical sensors, heavy metals, carbon nanoparticles, metal complexation, fluorescence quenching, fluorescence turn-on

## Abstract

A novel type of graphene-like nanoparticle, synthesized by oxidation and unfolding of C_60_ buckminsterfullerene fullerene, showed multiple and reproducible sensitivity to Cu^2+^, Pb^2+^, Cd^2+^, and As(III) through different degrees of fluorescence quenching or, in the case of Cd^2+^, through a remarkable fluorescence enhancement. Most importantly, only for Cu^2+^ and Pb^2+^, the fluorescence intensity variations came with distinct modifications of the optical absorption spectrum. Time-resolved fluorescence study confirmed that the common origin of these diverse behaviors lies in complexation of the metal ions by fullerene-derived carbon layers, even though further studies are required for a complete explanation of the involved processes. Nonetheless, the different response of fluorescence and optical absorbance towards distinct cationic species makes it possible to discriminate between the presence of Cu^2+^, Pb^2+^, Cd^2+^, and As(III), through two simple optical measurements. To this end, the use of a three-dimensional calibration plot is discussed. This property makes fullerene-derived nanoparticles a promising material in view of the implementation of a selective, colorimetric/fluorescent detection system.

## 1. Introduction

Research on nanostructured materials has recently produced noteworthy results in several fields, including biology, medicine, energy, and sensors [1,2,3,4,5,6]. In particular, graphene nanoparticles, also referred to as graphene oxide quantum dots (GOQDs), have demonstrated themselves to be a fluorescent nanomaterial with possible applications in chemical and biological optical sensing [7,8,9,10,11]. In fact, the stable blue/green fluorescent emission reported by several groups in GOQDs [12,13,14,15,16], regardless of the different synthesis methods, exhibited a fluorescence-quenching effect in the presence of certain heavy metal ions (Hg^2+^, Fe^3+^, Cu^2+^, Pb^2+^), generally attributed to the metal ion chelating with the different functional groups [17,18,19,20,21,22,23,24]. This property makes GOQDs promising for applications in optical sensors for the detection of heavy metals, which have long been known as highly toxic for human health above certain concentration values [25,26]. In the Guidelines for Drinking-Water Quality by World Health Organization (WHO) [27], for instance, the limit for arsenic and lead is 10 μg/L, for cadmium is 3 μg/L, while for copper it reaches up to 2 mg/L. In order to detect and measure heavy metal concentrations, potentially portable, fast, easy-to-use, and cheap optical methods are desirable, since the traditional techniques for standard laboratory analysis generally need expensive and heavy instrumentation which require highly skilled staff and time consuming procedures [28]. To this end, compared to other fluorometric materials, GOQDs seem to offer some distinct advantages that could offset a generally lower sensitivity [17]. In comparison to molecular fluorophores, for instance, GOQDs possess higher stability with respect to photobleaching and better biocompatibility, as it was demonstrated in several studies on bioimaging, with specific regard to live cell labelling and imaging [7,8,9,10,11,29,30]. Moreover, sensing properties have also been found in bare GOQDs, i.e., without a specific metal-oriented functionalization, differently from semiconductor quantum dots and metal nanoparticles, which need capping agents, and are therefore quite affected by the nature of surface states [3,31]. In fact, the intrinsic presence of oxygen-containing functional groups gives GOQDs a hydrophilic character that naturally stabilizes nanoparticle suspensions in aqueous media without any further functionalization [17]. However, few experimental studies have been performed, so far, in bare GOQDs, to assess the chance of practical applications of the sensing effect. In addition, the analysis of the literature shows variable and contradictory results, which also depend on the synthesis procedures. For instance, fluorescence quenching in the presence of Fe^3+^ ions was found by Wang et al. [18] in GO nanosheets prepared from graphite flakes. Differently, in a similar material [19], there was demonstrated selective sensitivity to Cu^2+^, and no response to other species, including Fe^3+^. In GOQDs synthesized with a bottom-up method, fluorescence quenching in the presence of Hg^2+^ was reported [20], whereas in GQDs synthesized from carbon fibers [22,24], an even more complex situation was observed, with multiple sensitivity to Co^2+^, Mn^2+^, Ni^2+^, and Cu^2+^. Finally, response to Cr^6+^ was reported by using commercially available GOQDs [23].

The differences in dimensions, content of defects and functional groups, depending on the different methods of preparation and starting compound for GOQDs, could account for the reported variety of results [32]. In any case, this situation is not helped by designing a single sensing system able to reliably differentiate between several metal species. With the aim of using a starting compound with a more defined structure and molecular weight, we have recently studied a novel type of graphene-like nanoparticle [33], synthesized by oxidation and cage-opening of C_60_ fullerene [34]. These unfolded fullerene quantum dots (UFQDs), showed reproducible fluorescence quenching in the presence of Cu^2+^, As(III), and Pb^2+^.

In the present work, the study has been extended to a larger number of cations since Cd^2+^, As(V), and Ca^2+^ have been added to the ensemble of As(III), Hg^2+^, Fe^2+^, Cu^2+^, Ni^2+^, Pb^2+^, Co^2+^, and Na^+^ previously investigated. This allowed us to observe that cadmium ions produce a remarkable *negative* quenching, that is a fluorescence enhancement, which had never been found in regular GOQDs. In addition, and more importantly, the usual measurements of fluorescence quenching have been integrated with accurate recording of the variations of the optical absorption spectra upon titration. This strategy allowed a peculiar combination of two calibration curves, which presents unique characteristics for each species. Time-resolved fluorescence lifetime measurements have been carried out in order to get more insight on the physical origin of the sensitivity to the different cations.

## 2. Materials and Methods

### 2.1. Materials

All chemicals, including C_60_ buckminsterfullerene (TERM USA, Fort Bragg, CA, USA), sodium hydroxide (Carlo Erba, Milano, Italy), sulphuric acid (99.9%, Sigma Aldrich, Milano, Italy), potassium permanganate (Sigma Aldrich), sodium nitrate (Sigma Aldrich), and hydrogen peroxide (Sigma Aldrich) were used as purchased without any further purification. For the measurements of sensitivity to metal ions, the metal salt solutions were prepared at the concentration of 200 µM in deionized water a few days before use, and stored at 5 °C to avoid degradation. That was particularly important in the case of salt containing arsenic(V), which tends to reduce to arsenic(III) [35]. The salt solutions were then diluted to the appropriate values of concentration for the measurements.

### 2.2. Synthesis of UFQDs

Unfolded fullerene quantum dots (UFQDs) were synthesized by using a modification of classical Hummer method [36], as previously reported [34]. The oxidation and opening of the C60 structure was possible thanks to strong oxidant agents and to temperature variations. The reaction was stopped with the addition of H_2_O_2_, and concentrated NaOH was used to acquire a solution at pH 8. The final product was dialyzed with a retained molecular weight of 2000 Da for two days to remove the extra acid and salt. After dialysis, the solution was diluted 1:5 with deionized water to obtain the stock solution. By drying the solution, the unfolded fullerene weight concentration of the stock solution was estimated to be about 0.2 mg/mL. In order to study and take account of the reproducibility of synthesis, the entire preparation procedure was repeated 10 times on different days over a 6 month period.

### 2.3. Instrumentation and Procedure for Detection of Heavy Metal Ions

Fourier transform infrared spectroscopy (FT-IR) data were obtained on powders by a Perkin Elmer Spectrum One spectrometer (Waltham, MA, USA). ^13^C-NMR spectra were recorded with a Bruker Avance 300 spectrometer (Bruker Corp., Billerica, MA, USA). The surface analyses were carried in an electronic spectrometer Escalab MkII (VG Scientific Ltd., London, UK) equipped with XPS and AES techniques. The Al Kα source was used for the photoemission and X-ray induced Auger spectroscopy, whereas a LEG200 electron gun was used for the AES. The photoemission spectra were collected at 50 eV pass energy in selected area mode A3 × 10 with a diameter of ≈3 mm, whereas the Auger spectra were registered at the pass energy of 100 eV, in order to increase the signal-to-noise ratio. All experimental data were processed by using the software Avantage v.5 (Thermo Fisher Scientific, Waltham, MA, USA). Experimental C KVV spectra were smoothed at least for 11 times by moving average routine with a width of 1.2 eV. Afterwards, these spectra were differentiated by using a width of 7 data points for the determination of D parameter [37]. Atomic force microscopy (AFM) was carried out in tapping mode (AC) with Si tip (spring constant around 40 N/m) on freshly cleaved Mica substrate after the deposition (and drying) of UFQD in aqueous solution. A digital pH meter (Hanna Instruments, Padova, Italy) was used to check the pH of the solutions. UV–vis absorption spectra were recorded with a Cary 50 spectrophotometer (Varian Inc., Palo Alto, CA, USA, USA) by using fused silica cuvettes with 10 mm optical path. Photoluminescence (PL) spectra were acquired on a laboratory set-up [38] equipped with an emission 25 cm monochromator (Cornerstone 260, Oriel Instruments, Stratford, CT, USA), specific excitation-rejection filters, and a R3896 photomultiplier (Hamamatsu Photonics Corporation, Bridgewater, NJ, USA), by using an optical length of 10 mm in a standard 90° geometry to reduce the effective sample length experienced by both the excitation and the emission light to 0.5 mm, approximately. In this way, it was possible to reduce artefacts in the PL quenching due to inner filter effects and transmittance variations [22]. The optical characterization of UFQDs was performed by using a 200 W continuous Hg(Xe) discharge lamp (Oriel Corp.) as optical source, with an excitation 25 cm monochromator (Photon Technology International, Inc., Birmingham, NJ, USA) and appropriate optical filters. The PL quenching by metal ions, instead, was characterized by using a 10 mW continuous wave LED at 300 nm (Thorlabs Inc., Newton, NJ, USA). The spectral response of the set up for PL detection was calibrated over the visible spectral range with a reference fluorophore solution. This enabled the fully correction of the emission curves. A spectral bandpass of 3 nm was used for both the excitation and emission monochromators. Fluorescence decay curves were recorded in a single photon counting spectrofluorometer (Fluoromax-4, Horiba, Irvine, CA, USA), and data were corrected both for dilution and for inner filter effects. The response to metal ions was characterized against a reference (blank) solution which was prepared by mixing 1.5 mL of the stock solution with 1.5 mL of deionized water. The measurements in the presence of metal ions were performed after adding 1.5 mL of the stock solution to the same volume of the metal–salt water solution at the appropriate concentration.

## 3. Results and Discussion

### 3.1. Morphological and Optical Characterization of UFQDs

**Figure 1:**
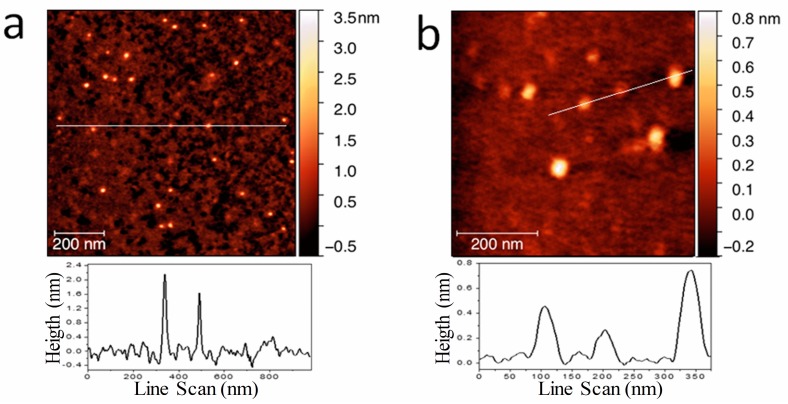

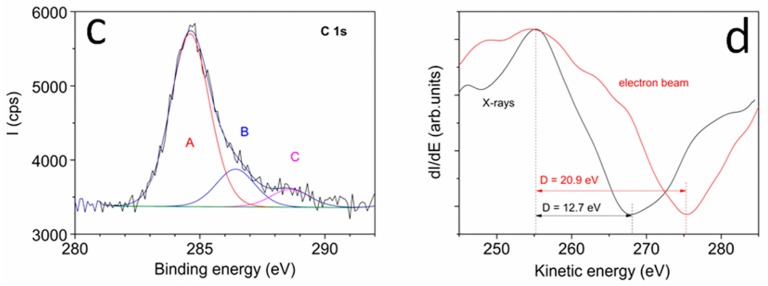
(**a**) Low-magnification and (**b**) high-magnification AFM image of unfolded fullerene quantum dots (UFQDs) deposited on the mica substrate and the height profile along the lines indicated in the images; XPS spectrum of C 1 s region (**c**) and Auger spectrum of C KVV region (**d**).

**Figure 2:**
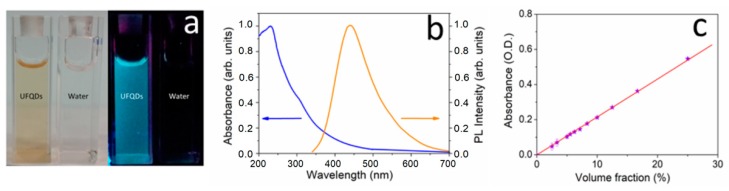
(**a**) Photograph of UFQD stock solution and deionized (DI) water taken under visible light (left) and 365 nm UV light (right); (**b**) Fluorescence and UV–vis absorption spectra of UFQDs stock solution; (**c**) Optical absorbance at λ_abs_ = 275 nm of the stock solution diluted at different volume fraction in deionized water.

AFM images of the dried stock solution (Figure 1a,b) showed particles with lateral dimensions of 15 nm, approximately, and height of about 0.5–2 nm, consistently with values observed in GO and graphene nanoparticles [39,40]. In particular, the lateral dimensions indicated that the aggregation of UFQDs occurred during solvent evaporation [34] (the tip convolution effect cannot exceed an enlargement more than 1–2 nm for the measured height). In fact, transmission electron microscopy analysis and dynamic light scattering measurements (DLS) previously performed on the same samples [33] (not reported here) showed the presence of a uniform dispersion of UFQDs nanoparticles with lateral dimensions ranging from 2 to 4 nm. The XPS spectrum of C 1s region reported in (Figure 1c) testified the prevalence of C–C bonds (component A) with binding energy (BE) = 284.6 eV [37]. Other two minor components of C 1s peak are attributed to C–O bonds, with BE = 286.4 eV, and carboxylic bonds with BE = 288.6 eV. From the spectra of C, the KVV region was acquired by using X-rays and electron beam (see Figure 1d), and the values of D parameter were determined to 12.7 and 20.9 eV, respectively. This change of the D parameter from the diamond-like value obtained with X-rays to graphitic one with electron beam indicates that the main configuration of C–C bonds corresponds to graphene [37,41]. The FT-IR and ^13^C NMR spectra (reported in Ref. [33]) presented similar features to those found in GOQDs, and the open-cage fullerene QDs [15,19,34,42,43]. In particular, the FT-IR spectrum showed the C=C vibration, that can be attributed to the presence of carboxylic acid and hydroxyl groups in the carbon nanoparticles. With regard to the optical properties, the stock solution was clear, with a slight brownish color, and did not show appreciable Tyndall effect with a He–Ne laser pointer, while a visible blue photoluminescence appeared under illumination with a 365 nm Wood’s lamp (Figure 2a). These characteristics have remained stable for at least one year at room temperature. The fluorescence and the absorption spectra (Figure 2b) showed the characteristic spectral features found in regular GOQDs synthesized either from graphite or from citric acid with a bottom-up procedure [17,18,19,20,21,22,23,24]. In particular, the UV–vis absorbance spectrum exhibited the typical well-resolved peak at 230 nm, generally attributed to the π–π* transition of the aromatic sp^2^ domains, and one more peak at ≈305 nm, which is due to the n–π* transition in C=O bonds of oxygen-containing functional groups [12,44]. It should be noted that FT-IR, XPS, and optical spectra of the present UFQDs are particularly similar to those found in so-called reduced graphene oxide (rGO), i.e., after having undergone a physical or chemical reducing process [45,46,47], thus, with a relatively low content of oxygen-containing functional groups.

This supposedly small number of functional groups, however, seems to have a low effect on the stability of UFQDs in water, since the absorbance of the as-prepared solution (after dialysis) at different degrees of dilution in deionized water showed a linear increase with increasing UFQD volume fraction (see Figure 2c). This indicates that possible concentration effects, due to aggregation or interparticle interactions, are negligible in the entire range of concentration, which is up to 10 times the concentration of the reference solution used for the detection of metal ions. Moreover, the solutions remained stable for months at any value of concentration. These findings, along with the results of DLS previously reported [33], confirmed the model of a monodisperse solution of non-interacting carbon nanosheets.

### 3.2. Diversified Optical Response of UFQDs to Heavy Metal Ions

**Figure 3:**
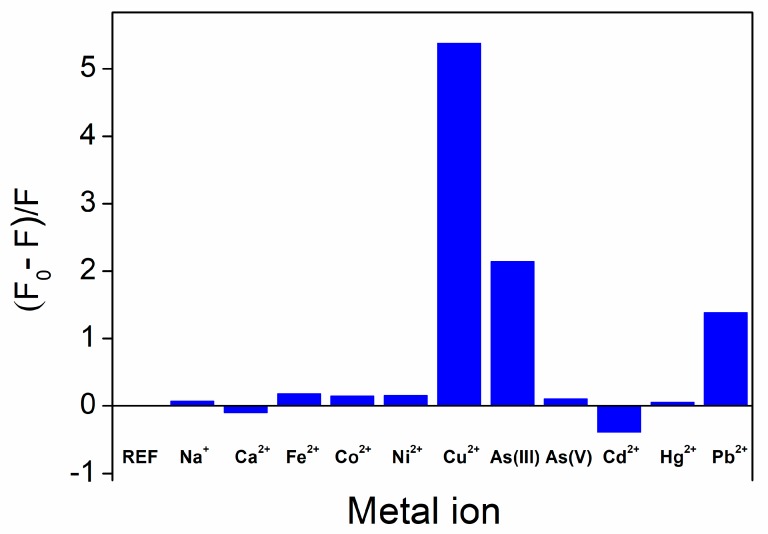
Fluorescence intensity ratio of the UFQD reference solution in the absence (REF) and presence of various metal ions at a concentration of 100 μM.

In line with the literature on regular GOQDs, the optical response of UFQD reference solution was first tested with regard to possible fluorescence variations against the addition of eleven different cations: 9 heavy metal ions, plus Ca^2+^ and Na^+^. Figure 3 shows the fluorescence quenching ratio F_0_/F-1 in the absence and presence of various ions at a concentration of 100 μM, extending the data reported in [33] to the results obtained with three new ions: Cd^2+^, Ca^2+^, and As(V). As can be seen, in addition to the typical fluorescence quenching occurring with Cu^2+^, Pb^2+^, and As(III), a somehow surprising fluorescence enhancement, or turn-on effect, was observed in the presence of cadmium ions. By recording the UV–vis absorption spectra of the reference solution, we found an even more complex situation, with different degrees of variation of absorbance after the addition of different species. In fact, the combination of the fluorescence quenching and absorption variation was a peculiar property of a specific metal ion. In the following, we will discuss these characteristics for each distinct species.

**Figure 4:**
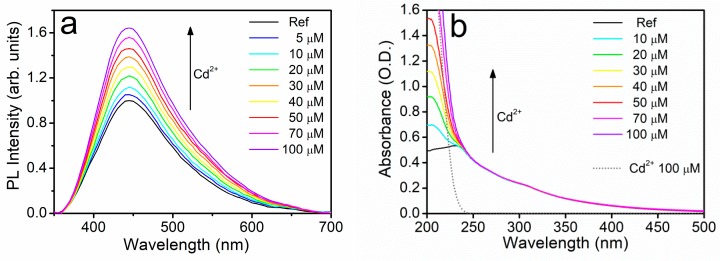
Emission (**a**) and optical absorption (**b**) spectra of the reference aqueous suspension of UFQDs in the presence Cd^2+^ at different concentrations. The absorbance of pure cadmium salt solution in water is also reported (dotted line).

First, we will present the remarkable fluorescence enhancement observed in the presence of Cd^2+.^ As displayed in Figure 4a, the progressive addition of cadmium ions to the reference UFQD solution produced the gradual increase of the fluorescence signal F without any appreciable change of the spectral profile. It should be noted that, to our knowledge, such a fluorescence turn-on effect had never been reported in GOQDs, whereas it is commonly observed in molecular fluorophores and semiconductor quantum dots [48,49,50]. At the same time, no variations were observed in the absorption spectra, as displayed in Figure 4b, apart from the progressive rise of the curve below 250 nm, which is clearly due to the increasing concentration of NO^3−^ ions coming from the added cadmium nitrate. This was checked by measuring the absorbance of a water solution containing only the cadmium salt at 100 μM of Cd^2+^ (dotted lines in Figure 4b). We verified the independence of the quenching effect from the anionic species by measuring a reasonably similar fluorescence turn-on effect with no variation of absorbance in cadmium chloride (see Figure S1 in Supporting Information). In addition, we performed an interference experiment for Cd^2+^ with some other relevant ions. While, as expected, the addition of cadmium ions to UFQD solution containing non-sensitizing ions such as Na^+^, Ca^2+^, Co^2+^, and Ni^2+^, led to minor variations in the fluorescence enhancement of cadmium, a great variation was found with Cu^2+^ (see Figure S1 in SI). In fact, cadmium only produced a very low partial recovery of the characteristic quenching effect of copper.

**Figure 5:**
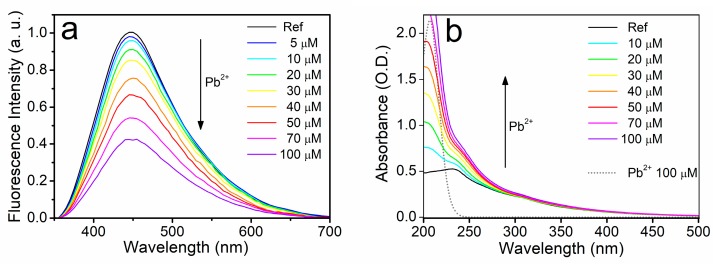
Emission (**a**) and optical absorption (**b**) spectra of the reference aqueous suspension of UFQDs in the presence of Pb^2+^ at different concentrations. The absorbance of pure lead salt solution in water is also reported (dotted line).

Quite differently from the case of Cd^2+^, the progressive addition of Pb^2+^ produced a typical fluorescence quenching effect, since the fluorescence signal F gradually decreased with the increasing ion concentration (see Figure 5a). This behavior resembles the typical fluorescence quenching effect reported in the literature on regular GOQDs [17,18,19,20,21,22,23,24]. As a further difference with respect to cadmium ions, Pb^2+^ also modified the absorption of UFQDs, producing a progressive increase of the absorbance in the wavelength range 250–340 nm (see Figure 5b), with an appreciable smearing of the peak at 300 nm. As discussed in the case of cadmium, this increase of absorbance cannot be attributed to the direct presence of the metal ions added to the solution. Instead, it is necessarily an effect of the interaction between metal ions and carbon layers. As will be discussed in Section 3.3, a static quenching mechanism, arising from the formation of ion–UFQD complexes, can account for this interaction.

The progressive addition of Cu^2+^ produced a similar response of UFQDs (data not shown here, see Ref. [33]), even though with a slightly higher quenching ratio and a lower increase of the absorption than those observed with Pb^2+^. Finally, the addition of As(III) induced a further different optical effect since the fluorescence intensity decreased with increasing metal ion concentration, while no variations were observed in the absorption spectra (see Ref. [33]). Interestingly, UFQDs discriminated As(III) from As(V), since the latter species did not induce any significant optical variation (see Figure 3).

**Figure 6:**
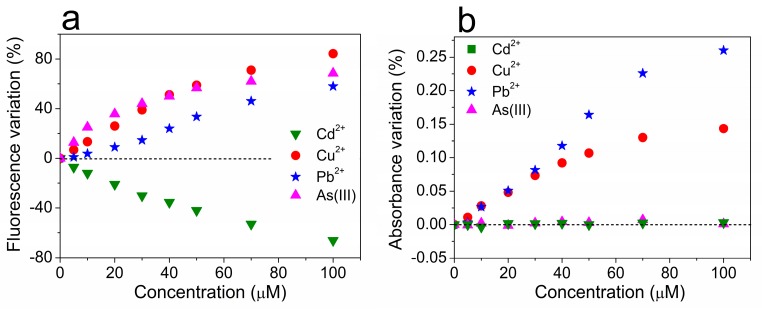
The percentage variation of the fluorescence intensity (**a**) and optical absorbance at λ_abs_ = 275 nm (**b**) of the reference aqueous suspension of UFQDs in the presence of Cd^2+^, Cu^2+^, Pb^2+^, and As(III) at various concentrations.

The diversification of response of UFQDs to the four sensitizing ions can be clearly seen in Figure 6, which shows the comparison of the different calibration curves obtained from the measurements reported above, and separately displayed for the variation of fluorescence intensity (Figure 6a) and of the optical absorbance (Figure 6b). 

In summary, three different types of optical response of UFQDs can be observed with regard to the variations of fluorescence and absorption spectra and they are reported in Table 1.

**Figure 7:**
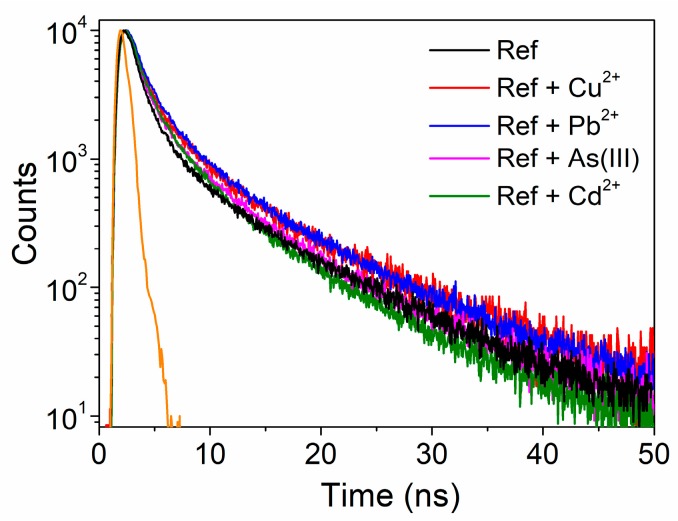
Fluorescence decay of UFQDs in the absence (black line) and presence (colored lines) of the metal ions (λ_exc_ = 280 nm). Instrumental response function is represented by the orange line.

Time-resolved fluorescence lifetime study can help understand the physical processes responsible for fluorescence and absorption variations. Figure 7 shows the fluorescence decays of UFQDs after a pulsed excitation at 280 nm in the absence and in the presence of the different metal ions at a concentration of 100 μM. All the decay curves were well fitted using tri-exponential functions having short (τ_1_), medium (τ_2_), and long (τ_3_) lifetime components, with respective weight coefficients A_i_ (see Table 2). These components can be related to optical transitions originating from different fluorescent sites of carbon nanoparticles [12,13,14,15,16,51]. For all the fluorescence-quenching species of the present study, i.e., Cu^2+^, Pb^2+^ and As(III), the quenched-fluorescence lifetimes were not decreased in comparison to reference, thus confirming the occurrence of static quenching through complexation of the metal ions, rather than dynamic quenching through collisional deactivation. In particular, Cu^2+^ and Pb^2+^ slightly increased the values of all the lifetime components, and mainly decreased the weight of the shortest components A_1_ and A_2_, thus resulting in an appreciably slower overall decay. Differently, As(III) evenly decreased the weight of all the components, whereas Cd^2+^ mainly increased the weight of τ_2_ and shortened the slowest component τ_3_, producing a peculiar faster decay in the long time region. We note that these three types of time responses paralleled the steady-state optical behavior described in Table 1. 

### 3.3. Possible Mechanisms for the Optical Response

**Scheme 1:**
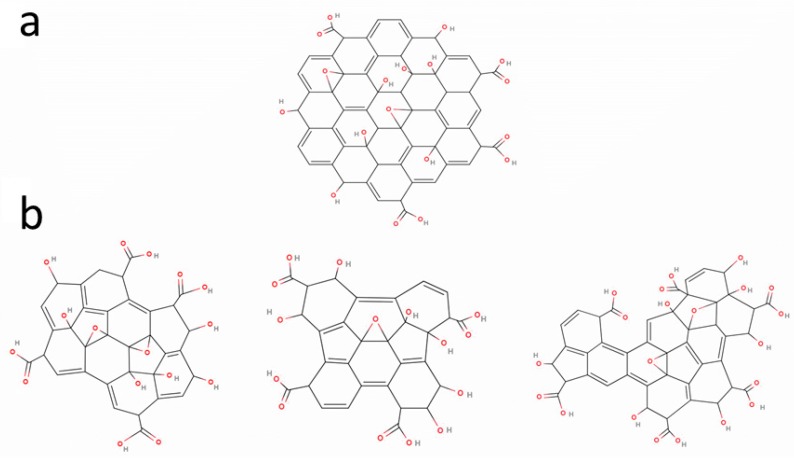
(**a**) Structure of graphene oxide (GO) nanoparticles compared with (**b**) possible structures of UFQD nanosheets.

The variations of fluorescence steady-state intensity, with substantially unvaried fluorescence lifetimes, and the modifications of absorption spectra in the range 250–340 nm can both be explained by the interaction of metal ions with UFQDs through formation of stable complexes. This mechanism had already been proposed for fluorescence quenching in regular GOQDs [20,21,22]. Moreover, several studies on GOQDs and GO have investigated the aggregation processes that occur via multiple complexation, i.e., with the metal ions binding more carbon layers together through coordination with the various oxygen-containing dangling groups [52,53,54,55,56]. As displayed in Scheme 1, GOQDs and UFQDs possibly have different lattice structure, but certainly share the presence of carboxyl, hydroxyl, and epoxy groups, as discussed in Section 3.1. Photoinduced electron transfer (PET) mechanisms are generally considered to play a significant role in the interaction of metal ions with the molecular orbitals of the functional groups [21,22,49]. Within this framework, the extensive literature on the metal adsorption by carbon nanomaterials [54,57,58,59,60,61] suggests that the three distinct types of optical responses observed in present UFQDs can be explained with combinations of the properties of the metal ions and the affinity with different sites or oxygen-containing groups [62].

The fluorescence quenching with increase of absorption, observed with Cu^2+^ and Pb^2+^, can be explained with the metal ions binding to UFQDs through the carboxyl groups located at the edges, possibly promoting an edge-to-edge aggregation of UFQDs [52,53,57,59,62], as displayed in Scheme S1a of Supporting Information. The different response observed after the addition of As(III) could be due to the fact that arsenic is known to bind with the surface of the carbon nanosheets, rather than the edge [63]. As a consequence, face-to-face stacking (see Scheme S1b) could occur, and this would quench fluorescence through radiationless decay mechanisms of the excited state [55], without altering the absorption spectrum. Finally, the noteworthy fluorescence enhancement observed with Cd^2+^ resembled the fluorescence turn-on effect frequently observed in molecular fluorophores after chelating this ion, and is referred to as “chelation-enhanced fluorescence” (CHEF). CHEF is generally attributed to a peculiar mechanism of interaction, involving PET processes of the fluorescent ligand [49]. Briefly, Cd^2+^ might coordinate with the basal surface of the carbon layers trough cation-π interaction [64] using the oxygen lone pair electrons of the carbonyl and epoxy groups that, in the absence of the cadmium ion, would instead fill the empty states left by the optical excitation and reduce the radiative recombination rate (see Scheme S2 in SI). Therefore, by immobilizing the lone pair electrons, Cd^2+^ increases the recombination rate and the fluorescence intensity of UFQDs. This model is consistent with the decrease of τ_3_ reported in Section 3.1 and face-to-face stacking, similar to As(III).

### 3.4. Comparison with Regular GOQDs, LODs, and Three-Dimensional Calibration Diagram

More investigations are required to confirm the hypothesis made in the previous paragraph about the origin of the different optical behaviors of UFQDs. However, we note that sensitivity of a single solution to more metal ions was previously observed only in GOQDs synthesized from carbon fibers [22,24] which showed fluorescence quenching to seven metal ions, including Cu^2+^, Ni^2+^, and Co^2+^. In addition, it should be pointed out that neither the response to As(III) nor the remarkable fluorescence enhancement observed with the addition of Cd^2+^ have ever been reported in regular GOQDs. In the present experiment, from the calibration curves of Figure 6 in the low concentration limit, the following limits of detection (LODs) can be estimated, according to the IUPAC definition [65]: 350 nM, 1 μM, 500 nM, and 350 nM for Cu^2+^, Pb^2+^, As(III), and Cd^2+^, respectively. While these values are in line with those reported in the literature for regular GOQDs [18,19,20,22,24], only the LOD for Cu^2+^ is well below the current limit of 30 μM suggested by WHO for drinking water, and thus, meets the fundamental requirement for a real sensing material. Conversely, the present values are definitely much higher than the limits of 50 nM for lead and 14 nM for arsenic. In this regard, it should be noted that neither regular GOQDs nor present UFQDs reach the LODs in the picomolar range achieved by some specifically engineered molecular chromophores [31] which, however, rarely exhibit multiple sensitivity to different ions. With specific regard to Cd^2+^, large fluorescence turn-on effects can occur in molecular chromophores due to PET mechanisms interacting with rearrangements of the molecular structure [31], and this can lead to LODs as low as 0.1 pM [66]. However, in most of the molecular sensors reported in the literature, values in the nanomolar range, reasonably comparable to the present ones, are reported [48,67]. On the other hand, as discussed in the introduction, few experimental works have been carried out so far on the peculiar properties of carbon nanomaterials, i.e., the multiple sensitivity of fluorescence quenching with simultaneous variation of absorbance.

**Figure 8:**
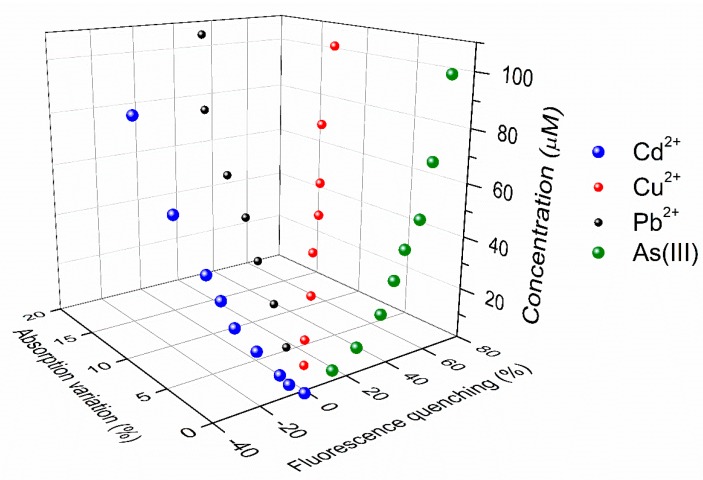
In the three-dimensional graph, the concentration of a specific ionic species is reported as a function of the fluorescence variation and the relative increase of absorbance it produces on UFQDs.

With regard to the selectivity, we note that while the calibration curves of Figure 6 clearly show the variety of response of UFQDs towards the different ions, they do not directly help identify the distinct species. For instance, at low concentration, the variation of absorption in the presence of Pb^2+^ is practically indistinguishable from that of Cu^2+^, while both Cd^2+^ and As(III) steadily produce no variation at all (Figure 6b). Similarly, in the range of concentrations from 30 to 50 μM, the fluorescence quenching produced by As(III) almost equals that given by Cu^2+^ (Figure 6a). However, the two sets of measurements can be combined together to provide the more informative three-dimensional calibration diagram (3DCD) of Figure 8. In 3DCD, the results of titration measurements are reported in a 3D space, with the Z axis representing the concentration of each ion while the X and the Y axes report the variation of fluorescence and absorption, respectively. We note that in this space, the data referring to Cd^2+^ and As(III) are in the XZ plane, since these two ionic species do not produce any variation of absorption. On the other hand, the points relative to cadmium are in the negative semispace of fluorescence quenching. More importantly, datasets of different ions do not cross each other, differently from what occurs in Figure 6, thus demonstrating that a certain species at a given concentration produces a specific couple of values of fluorescence quenching and variation of absorption. In other words, through this three-dimensional plot, the measurement of a certain pair of values of the two optical quantities uniquely determines the concentration of a specific metal ion, as shown with two practical examples in Figure S2 of SI. This property of UFQDs, in principle, can give selectivity to the process of ion detection, and is a promising feature for developing a multiple selective optical detection of heavy metals in aqueous media.

## 4. Conclusions

In conclusion, by recording the variation induced in both fluorescence and optical absorption spectra, we have found that UFQDs exhibited a more complex and diversified behavior than the fluorescence quenching already reported in the literature for regular GOQDs. In fact, different types of optical response were detected to four ions: Pb^2+^, Cu^2+^, Cd^2+^, and As(III). In addition to different degrees of fluorescence quenching in the presence of Pb^2+^, Cu^2+^, and As(III), appreciable variations of absorption were also revealed with Pb^2+^ or Cu^2+^, whereas a remarkable fluorescence enhancement was detected with Cd^2+^. This last result, in particular, represented the first evidence of fluorescence turn-on effect in non-functionalized carbon-based sensing. Fluorescence lifetime measurements and pH dependence of fluorescence variations confirmed that, in all cases, the response is due to metal ions forming stable complexes with carbon layers, possibly through distinct type of chemical bonds and binding sites. More investigations, however, are needed to understand how these processes produce differences in the optical outcome. In view of applications to real sensing systems, the LODs for Pb^2+^, Cd^2+^, and As(III) are definitely too high in comparison with the current limits of WHO, and further steps are needed to meet this fundamental requirement. To this end, we think that the noteworthy property of UFQDs, i.e., the diversification of the optical response towards different ions, deserves more studies in view of the chance of differentiating distinct species through fluorescence and absorption measurements. In principle, this characteristic is the key for the implementation of a selective multiple optical sensor.

## Figures and Tables

**Table 1:** The different types of optical response with regard to fluorescence intensity and absorbance in the range 250–340 nm of UFQD reference solution in the presence of heavy metal ions.

Sensitizing Ion	Fluorescence Variation	Absorbance Variation
Cu^2+^, Pb^2+^	decrease	increase
As(III)	decrease	steady
Cd^2+^	increase	steady

**Table 2:** Values of the lifetime components τ_i_ and the respective weight constants A_i_ of the three-exponential fit of the fluorescence decay in the absence (REF) and the presence of the different metal ions. A_i_ values are normalized to those of the reference solution.

	τ_1_ (ns)	A_1_	τ_2_	A_2_	τ_3_	A_3_
REF	0.557	1	2.404	1	10.30	1
Cu^2+^	0.648	0.30	3.06	0.35	11.35	0.5
Pb^2+^	0.685	0.68	2.94	1.05	10.69	1
As(III)	0.602	0.45	2.99	0.55	10.30	0.6
Cd^2+^	0.629	1.37	2.74	1.90	9.55	1.5

## Supplementary Materials

The following are available online at http://www.mdpi.com/1424-8220/18/5/1496/s1, Figure S1: Interference of fluorescence quenching ratio of UFQD reference solution with cadmium in the presence of various metal ions:, Scheme S1: Possible mechanisms of aggregation of UFQDs; rom carbonyl and hydroxyl groups, Scheme S2: Schematic of the chelation enhanced fluorescence (CHEF) process; Figure S2: Two examples of applications of the three-dimensional calibration diagram.
