# Supplementary material for: Discriminating between Different Heavy Metal Ions with Fullerene-Derived Nanoparticles
Source: Sensors (Basel). 2018 May 10;18(5):1496. doi: 10.3390/s18051496 (PMC5982591; doi:10.3390/s18051496)
Supplement: Supplementary file 1 [file sensors-18-01496-s001.pdf]

Supporting Information

Discriminating between Different Heavy Metal Ions with Fullerene-Derived Nanoparticles

Erica Ciotta, Paolo Prosposito, Pietro Tagliatesta, Chiara Lorecchio, Lorenzo Stella, Saulius Kaciulis, Peiman Soltani, Ernesto Placidi and Roberto Pizzoferrato

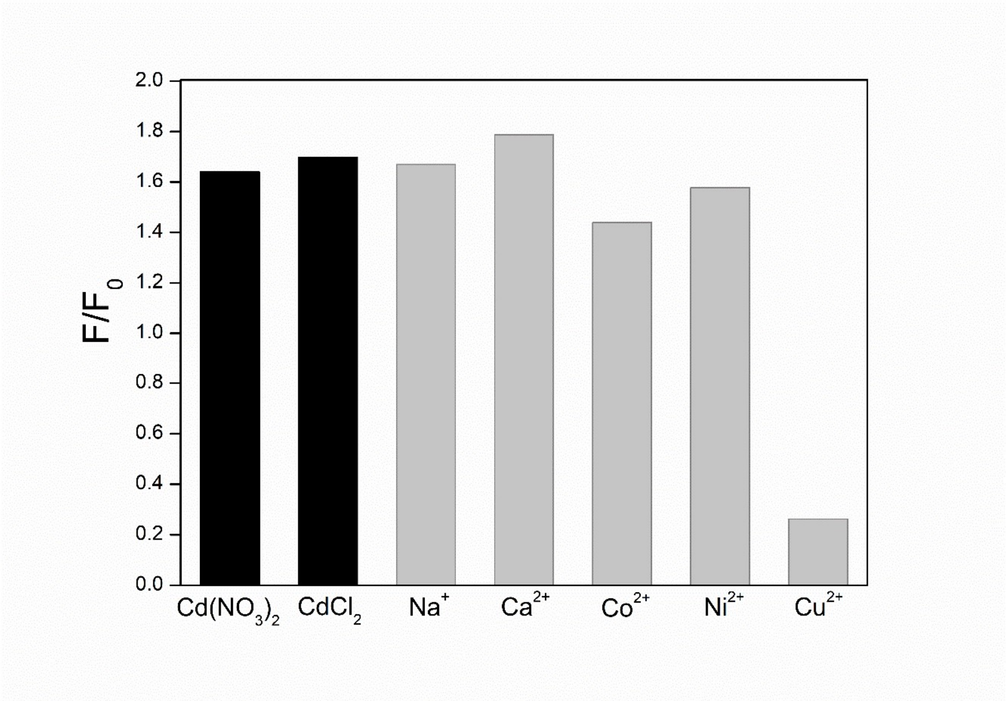

Figure S1. Fluorescence quenching ratio of UFQD reference solution in the presence of various metal ions: (black bars) in the presence of different salts of cadmium with a concentration of 100 μM of Cd^{2+}; (grey bars) in the presence of various metal ions at 100 μM, followed by 100 μM of Cd^{2+}.

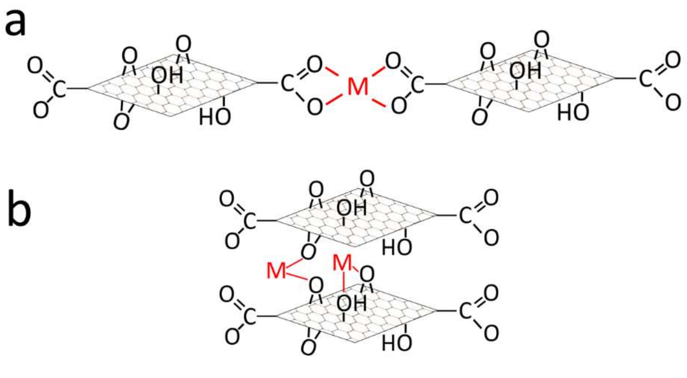

Scheme S1. Possible mechanisms of aggregation: (**a**) edge-to-edge through chelating carboxyl groups; (**b**) face-to-face staking through either weak alkoxide or dative bonds from carbonyl and hydroxyl groups.

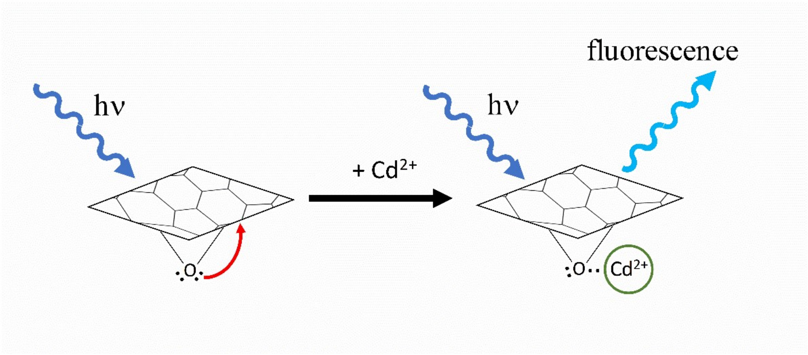

Scheme S2. Schematic of the chelation enhanced fluorescence (CHEF) process due to the chelation of a Cd^{2+} ion which immobilizes the oxygen electron lone pair.

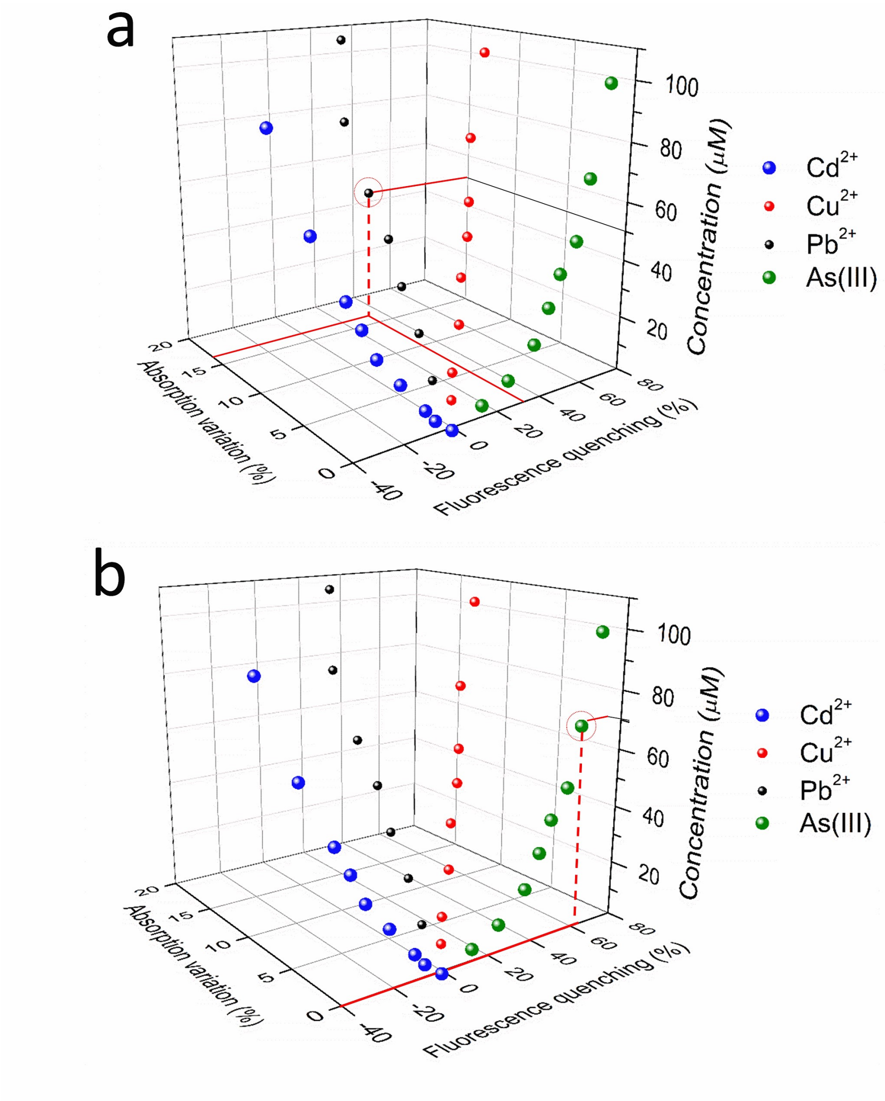

Figure S2. Two examples of applications of the three-dimensional calibration diagram. In (**a**) a fluorescence quenching by 33% and an increase of absorbance by 16% (marked by the red solid lines) uniquely correspond to 50 μM of Pb^{2+}; in (**b**) a 62% variation of fluorescence intensity with no variation of absorbance uniquely correspond to 70 μM of As(III).
